# Complete Genome Sequences of Two Acetylene-Fermenting *Pelobacter acetylenicus* Strains

**DOI:** 10.1128/genomeA.01572-16

**Published:** 2017-02-09

**Authors:** John M. Sutton, Shaun M. Baesman, Janna L. Fierst, Amisha T. Poret-Peterson, Ronald S. Oremland, Darren S. Dunlap, Denise M. Akob

**Affiliations:** a Department of Biological Sciences, The University of Alabama, Tuscaloosa, Alabama, USA; b U.S. Geological Survey, National Research Program-Western Branch, Menlo Park, California, USA; c U.S. Geological Survey, National Research Program-Eastern Branch, Reston, Virginia, USA

## Abstract

Acetylene fermentation is a rare metabolism that was serendipitously discovered during C_2_H_2_-block assays of N_2_O reductase. Here, we report the genome sequences of two type strains of acetylene-fermenting *Pelobacter acetylenicus*, the freshwater bacterium DSM 3246 and the estuarine bacterium DSM 3247.

## GENOME ANNOUNCEMENT

The fermentation of acetylene (C_2_H_2_) to ethanol and acetate is a rare metabolic pathway (1). Only three well-characterized microbial strains, all within the *Pelobacter* genus, can grow on C_2_H_2_ via acetylene hydratase (AH), a W-containing, exothermic, and low-redox-potential enzyme (1, 2). However, little is known about the regulatory controls and evolution of this metabolism.

Here, we present complete genome sequences of two acetylene-fermenting isolates, *P. acetylenicus* DSM 3246 and DSM 3247. Both strains were obtained from the DSMZ (Deutsche Sammlung von Mikroorganismen und Zellkulturen, Braunschweig, Germany). Freshwater strain DSM 3246 was grown in DSMZ medium 298 using 0.01 mM acetoin (CH_3_COCHOHCH_3_). Estuarine strain DSM 3247 was grown in DSMZ medium 293 using either acetoin or C_2_H_2_ (1.5%).

The genomes were sequenced at the University of California at Davis Genome Center (Davis, CA USA) using a Pacific Biosciences (PacBio) single-molecule real-time (SMRT) RSII instrument (Pacific Biosciences of California, Inc., Menlo Park, CA). Briefly, cultures were grown to high density and pelleted by centrifugation. Genomic DNA was extracted from multiple pellets with the DNeasy blood and tissue kit (Qiagen, Valencia, CA, USA), according to the manufacturer’s instructions but modifying the protocol by inverting in place of vortexing to reduce DNA shearing. DNA from multiple pellets was concentrated using either the Power Clean Pro kit (Mo Bio Laboratories, Inc., Carlsbad, CA, USA) or the Zymo Spin genomic DNA (gDNA) concentrator (Zymo Research Corporation, Irvine, CA, USA).

A sequencing-ready library with 10-kb inserts was prepared for each strain and sequenced with C4 chemistry on one SMRT cell. Sequencing of DSM 3246 produced 71,385 sequence reads, with an *N*_50_ size of 19,001 bp, and sequencing of DSM 3247 produced 62,650 sequence reads, with an *N*_50_ size of 18,239 bp. The genome of *P. acetylenicus* DSM 3246 was assembled into a 3,192,352-bp circular chromosome with 57.4% G+C content (222× coverage) and a 13,658-bp circular plasmid with 57.4% G+C content (36× average coverage) using the Hierarchical Genome Assembly Process (HGAP), which includes polishing with Quiver (3). The genome of *P. acetylenicus* DSM 3247 was assembled into a 3,176,363-bp circular chromosome (110× average coverage) with 57.4% G+C content, using the same procedure.

Annotation was performed using the Prokaryotic Genome Annotation Pipeline (PGAP) from the National Center for Biotechnology Information (NCBI) (4). DSM 3246 and DSM 3247 contained 2,976 and 2,914 genes, respectively. Functional annotation identified 2,774 coding genes, 16 rRNAs, 53 tRNAs, five noncoding RNAs, and 132 pseudogenes in DSM 3246. DSM 3247 contained 2,805 coding genes, nine rRNAs, 51 tRNAs, five noncoding RNAs, and 44 pseudogenes. Both DSM 3246 and DSM 3247 contained one type I clustered regularly interspaced short palindromic repeat (CRISPR) array.

Gene sequences for the five enzymes necessary for the fermentation of acetylene to ethanol and acetate (AH, aldehyde dehydrogenase, alcohol dehydrogenase, phosphate acetyltransferase, and acetate kinase) were annotated in both strains. DSM 3246 and DSM 3247 each contained one copy of the *ahy* gene encoding AH. The DSM 3246 *ahy* is 100% identical on the amino acid level to that of DSM 3247. Comparative analyses of these and other *Pelobacter* species may provide insight into the regulation and evolution of acetylene fermentation.

### Accession number(s).

Genomes are available from the NCBI GenBank database under BioProject PRJNA319824 and GenBank accession numbers CP015455 (DSM 3246 genome), CP015456 (DSM 3246 plasmid), and CP015518 (DSM 3247 genome).
